# Supplementary material for: Eating habit patterns may predict maximum occlusal force: A preliminary study
Source: PLoS One. 2022 Feb 15;17(2):e0263647. doi: 10.1371/journal.pone.0263647 (PMC8846518; doi:10.1371/journal.pone.0263647)
Supplement: S1 Table — (DOCX) [file pone.0263647.s001.docx]

**Eating habit patterns may predict maximum occlusal force: a preliminary study**

Masahiro Okada^1^*, Kosuke Okada^2^, and Masayuki Kakehashi^3^

^1^Department of Food and Dietetics, Hiroshima Bunka Gakuen Two-Year College, Hiroshima, Japan

^2^Department of Internal Medicine COOP Saeki Hospital, 3-11-29 Yahata-higashi, Saeki-ku, Hiroshima, Japan

^3^Graduate School of Biomedical & Health Sciences, Hiroshima University, Hiroshima, Japan

*Corresponding author: [okada@hbg.ac.jp](mailto:okada@hbg.ac.jp) (MO)

**S1 Table. Eating Habit Questionnaire.**

| **Eating habit item** | **Question** | **Answers** |
| --- | --- | --- |
| 1. **Breakfast habits** | **Are you in the habit of eating breakfast?** | **Skip sometimes　　　　　Every day** |
| 1. **Always eat at a fixed time** | **Do you usually eat at fixed times?** | **Yes　　　　　　　 No** |
| 1. **Number of meals per day (including snacks)** | **How many meals do you eat a day**  **(including snacks)?** | **meals/day** |
| 1. **Amount eaten** | **How much food do you usually eat?** | **Small　　　　 Medium　　　　 Large** |
| 1. **Eating speed** | **How quickly do you eat?** | **Slow　　　　　 Fast** |
| 1. **Chew food well** | **Do you chew your food well?** | **Yes　　　　　　　 No** |
| 1. **Eat until full** | **Do you eat until you are full?** | **Yes　　　　　　　 No** |
| 1. **Think about the nutritional balance of the meal** | **Do you pay attention to the nutritional balance of your meal?** | **Yes　　　　　　　 No** |
| 1. **Many likes and dislikes** | **Do you have many food likes and dislikes?** | **Yes　　　　　　　 No** |
| 1. **Eat for stress relief** | **Does eating relieve your stress?** | **Yes　　　　　　　 No** |
| 1. **Eat with others or alone (including family)** | **Are you always with someone when you eat (including family)?** | **Alone　　　Sometimes eat with others　　　Always eat with others** |
| 1. **Conversation when eating** | **Do you have a conversation when eating?** | **No conversation　　Sometimes conversation　　Always conversation** |
