# Supplementary material for: Eating habit patterns may predict maximum occlusal force: A preliminary study
Source: PLoS One. 2022 Feb 15;17(2):e0263647. doi: 10.1371/journal.pone.0263647 (PMC8846518; doi:10.1371/journal.pone.0263647)
Supplement: S2 Table — (DOCX) [file pone.0263647.s002.docx]

**Eating habit patterns may predict maximum occlusal force: a preliminary study**

Masahiro Okada^1^*, Kosuke Okada^2^, and Masayuki Kakehashi^3^

^1^Department of Food and Dietetics, Hiroshima Bunka Gakuen Two-Year College, Hiroshima, Japan

^2^Department of Internal Medicine COOP Saeki Hospital, 3-11-29 Yahata-higashi, Saeki-ku, Hiroshima, Japan

^3^Graduate School of Biomedical & Health Sciences, Hiroshima University, Hiroshima, Japan

*Corresponding author: [okada@hbg.ac.jp](mailto:okada@hbg.ac.jp) (MO)

| **S2 Table.** **Relationships between Maximum Occlusal Force and Body Composition.** | |
| --- | --- |
| Body composition | β (*P*) |
| Body fat percentage (%) | 0.168 (0.480) |
| Muscle mass (kg) | 0.367 (0.035) |

Analysis was made after adjusting for age and body mass index.

*P* values are shown in parentheses.

β*,* standardized regression coefficient
