# Supplementary material for: Eating habit patterns may predict maximum occlusal force: A preliminary study
Source: PLoS One. 2022 Feb 15;17(2):e0263647. doi: 10.1371/journal.pone.0263647 (PMC8846518; doi:10.1371/journal.pone.0263647)
Supplement: Attachment — Submitted filename: Review comments.docx [file pone.0263647.s004.docx]

**Manuscript title:** Eating habit patterns may help predict maximum occlusal force in

healthy young women: a preliminary study.

This is an interesting topic and I would like to thank the authors for the great effort and time they spend on this paper. However, I have some concerns and hope the authors will address them before recommending this manuscript for publication.

**Title:** The title is somewhat narrative. Please rewrite it to make it more vital, concise, attract the reader, and come to the point.

**Introduction:**

1. The introduction is too long.
2. You should start with a brief introduction of the topic, then write the body of the introduction by focusing on the previous studies related to your topic. After that tell the readers why you are conducting this study and why your research is important, and finally, conclude with your aims and objectives.

**Methos:**

1. Too long and repetitive
2. Please provide clear inclusion and exclusion criteria at the beginning of this section.
3. Subjective questionnaire about eating habits: Give a short description of the questionnaire. You needn’t write every single question in this section. You can add the questionnaire at the end of this paper as an index or supplementary file.

**Discussion:**

1. The discussion is also too long.
2. In lines from 251 to 255, you wrote ‘’ there was a significant but not strong relationship between some eating habits…… maximum occlusal force in healthy young women’’. Which eating habits were significant? Don’t force the reader to search about these significant habits across the manuscript. Please add it.
3. Lines 259 to 277 are repetitive to the introduction section. Please delete it.
4. In lines 278 to 280, you wrote ‘’ Additionally, we created an original simple eating habit questionnaire that excluded the factors of dietary ……. investigations of maximum occlusal force.’’ My question is, Have you tried to validate your questionnaire before commencing this study or not?

**Conclusion:**

- Please provide an accurate and concise conclusion that coincides with that in the abstract.

**References:**

There are 47 references. I advise the authors to reduce it as much as they can by selecting the most relevant ones.
